# WWL70 protects against chronic constriction injury-induced neuropathic pain in mice by cannabinoid receptor-independent mechanisms

**DOI:** 10.1186/s12974-017-1045-9

**Published:** 2018-01-08

**Authors:** Jie Wen, Melissa Jones, Mikiei Tanaka, Prabhuanand Selvaraj, Aviva J. Symes, Brian Cox, Yumin Zhang

**Affiliations:** 1 Department of Anatomy, Physiology and Genetics, Uniformed Services University of the Health Sciences, 4301 Jones Bridge Road, Bethesda, MD 20814 USA; 2 Department of Pharmacology and Molecular Therapeutics, 4301 Jones Bridge Road, Bethesda, MD 20814 USA; 3 Neuroscience Program, Uniformed Services University of the Health Sciences, 4301 Jones Bridge Road, Bethesda, MD 20814 USA

**Keywords:** Endocannabinoids, ABHD6, Chronic constriction injury, Neuroinflammation, Cyclooxygenase-2, Prostaglandin E synthase, PGE_2_, Neuropathic pain

## Abstract

**Background:**

Targeting the endocannabinoid system has emerged as an effective strategy for the treatment of inflammatory and neurological diseases. Unlike the inhibition of the principal 2-arachidonyl glycerol (2-AG) hydrolytic enzyme monoacylglycerol lipase (MAGL), which leads to 2-AG overload and cannabinoid receptor desensitization, selective inhibition of the minor 2-AG hydrolytic enzyme alpha, beta-hydrolase domain 6 (ABHD6) can provide therapeutic benefits without producing cannabimimetic side effects. We have shown that inhibition of ABHD6 significantly reduces neuroinflammation and exerts neuroprotection in animal models of traumatic brain injury and multiple sclerosis. However, the role of ABHD6 inhibition on neuropathic pain has not been explored.

**Methods:**

Neuropathic pain was induced by chronic constriction injury (CCI) of the mouse sciatic nerve and examined by Hargreaves and Von Frey tests. Activation of inflammatory cells and the production of cytokines and chemokines in the spinal cord dorsal horn, dorsal root ganglion (DRG), and sciatic nerve were assessed by qRT-PCR, enzyme-linked immunosorbent assay (ELISA), and immunohistochemistry. The levels of 2-AG and arachidonic acid (AA) in sciatic nerve were quantified by liquid chromatography coupled with tandem mass spectrometry (LC-MS/MS).

**Results:**

Treatment with the selective ABHD6 inhibitor WWL70 significantly alleviated CCI-induced thermal hyperalgesia and mechanical allodynia. Microglia activation, macrophage infiltration, and the production of nociceptive mediators were reduced in the ipsilateral lumbar spinal cord dorsal horn, DRG, and sciatic nerve of WWL70-treated animals. The diminished cytokine and chemokine production is likely due to the inhibitory effect of WWL70 on NF-κB phosphorylation. Surprisingly, the anti-nociceptive and anti-inflammatory effects of WWL70 were not reversed by addition of the cannabinoid receptor antagonists. Treatment with WWL70 did not alter the levels of 2-AG, AA, and the phosphorylation of cytosolic phospholipase A_2_ (cPLA_2_), but significantly reduced the production of prostaglandin E_2_ (PGE_2_) and the expression of cyclooxygenase-2 (COX-2) and prostaglandin E synthase-2 (PGES2) in the injured sciatic nerve.

**Conclusions:**

This study reveals a novel mechanism for the antinociceptive effect of the 2-AG catabolic enzyme ABHD6 inhibitor WWL70. Understanding the interaction between endocannabinoid and eicosanoid pathways might provide a new avenue for the treatment of inflammatory and neuropathic pain.

## Background

Neuropathic pain is a complex, chronic, and debilitating pain state that significantly worsens a patient’s quality of life [1]. Although maladaptive neuronal processes most likely trigger the onset of neuropathic pain, increasing evidence points to the crucial role of non-neuronal cells, such as immune cells, astrocytes, and microglia/macrophages, in the induction and maintenance of pain symptoms [2, 3]. Therefore, targeting the neuron-glial interaction or the neuroimmune interface is a suitable strategy for developing therapeutics to treat neuropathic pain.

Targeting the endocananbinoid system in both neurons and glial cells can alleviate inflammatory and neuropathic pain [4]. This system is composed of cannabinoid type 1 (CB1) and type 2 (CB2) receptors, the endogenous ligands anandamide (AEA) and 2-arachidonyl glycerol (2-AG), and enzymes for their synthesis and hydrolysis [5]. AEA and 2-AG are present in key regions involved in detection, relay, and integration of nociceptive inputs, such as the skin, dorsal root ganglion (DRG), spinal cord dorsal horn, and periaqueductal gray [6]. AEA preferentially binds to CB1R located primarily in neurons and is a weak agonist for CB2R mainly expressed in glia and immune cells; 2-AG is a full agonist for both CB1 and CB2 receptors [7, 8]. Following the induction of inflammatory and neuropathic pain, the levels of AEA, 2-AG, and the cannabinoid receptors are increased in spinal cord and DRG [9, 10], suggesting endocannabinoid signaling might modulate the initiation and propagation of pain. Consistently, blockage of 2-AG and AEA degradation attenuates mechanical allodynia and thermal hyperalgesia in several inflammatory and neuropathic pain animal models [11–15].

Despite the demonstrated potency of MAGL inhibitors in the management of neuropathic pain, chronic or complete inhibition of MAGL can cause 2-AG overload, CB1R desensitization, and behavioral tolerance [16]. Thus, incomplete inhibition of MAGL is thought to be more clinically useful to promote 2-AG signaling [17]. Alternatively, inhibition of the minor 2-AG hydrolytic enzyme α/β-hydrolase domain 6 (ABHD6) may possess a better therapeutic efficacy, given that its inhibition can elevate 2-AG levels to the therapeutic threshold without producing side effects [18]. Studies from our lab and others have shown that ABHD6 inhibition has anti-inflammatory and neuroprotective effects in animal models of inflammation, traumatic brain injury, and multiple sclerosis by enhancement of cannabinoid signaling and reduction of eicosanoid signaling [19–21].

An increasing number of studies have implicated microglia and astrocytes in the development, spread, and potentiation of neuropathic pain [22]. The production and release of inflammatory mediators, such as interleukin-1β (IL-1β), IL-6, tumor necrosis factor alpha (TNF-α), and prostaglandin E_2_ (PGE_2_) from activated microglia, lead to activation of adjacent astrocytes [23], which then act in concert to excite nociceptive neurons causing persistent pain [3]. We recently reported that the ABHD6 inhibitor WWL70 suppressed PGE_2_ production and the expression of its synthetic enzymes cyclooxygenase-2 (COX-2) and prostaglandin E synthase (PGES) in reactive microglia [24]. In agreement with these results, we find in this study that WWL70 significantly reduced neuropathic pain through interference with arachidonic acid (AA) metabolism and PGE_2_ production rather than inhibition of 2-AG breakdown.

## Methods

### Materials

The ABHD6 inhibitor WWL70, the CB1R antagonist AM281, and the CB2R antagonist AM630 were purchased from Cayman Chemicals (Ann Arbor, MI). All other chemicals and reagents were purchased from Sigma (St. Louis, MO), unless stated otherwise.

### Animals

Male 8- to 10-week-old C57BL/6J mice were purchased from the Jackson Laboratory (Bar Harbor, ME). Animal care and experimental procedures were carried out in accordance with NIH guidelines and approved by the Uniformed Services University Institutional Animal Care and Use Committee.

### Chronic constriction injury of the sciatic nerve

The surgical procedure for chronic constriction injury of the sciatic nerve was performed as described previously [25]. Mice were anesthetized with isoflurane (3.5% for induction and 2.0% for maintenance), and the mid to lower back of the animals along with the dorsal left thigh were shaved and cleaned with iodine and 75% ethanol. Using aseptic procedures, the common sciatic nerve was exposed at the mid-thigh level by blunt dissection. Under a dissection microscope, a nerve segment of 5 mm long was separated from the surrounding tissues. Two ligatures of 6–0 sterile silk, spaced 1.0 to 1.5 mm apart, were placed around the sciatic nerve. The ligatures were tied until they just constricted the diameter of the nerve, and a brief twitch was observed in the respective hind limb [26]. In sham-operated mice, the sciatic nerve was isolated and exposed without ligation. The muscles and skins were closed with sutures.

### Behavioral assessment of nociceptive behavior

#### von Frey test

Mechanical allodynia induced by loose ligation of the sciatic nerve was assessed with the “von Frey” test, as described [27]. The level of allodynia was determined by testing the withdrawal reflex to tactile stimuli with von Frey filaments of varying thickness. Mechanical thresholds were determined by the “Up-Down” method [28]. Mice were placed in a Plexiglas cage with mesh metal flooring and allowed to acclimate for 30 min before testing. A series of calibrated von Frey filaments (Stoelting, Inc., Wood Dale, IL) with logarithmically incremental stiffness ranging from 2.44 to 4.31 were applied to the mid-plantar surface of the hind paws. Each hair was presented perpendicularly against the paw, with sufficient force to cause slight bending, and held for 3 s. A positive response was noted if the paw was sharply withdrawn. Flinching immediately upon removal of the hair was also considered a positive response. In case of ambiguous response such as ambulation, the stimulus was repeated.

#### Hargreaves test

Thermal escape latency was determined using a Hargreaves type thermal escape testing system (Plantar Analgesia Meter, IITC Life Science Inc., Woodland Hills, CA). The same groups of animals used for von Frey test were placed individually in Plexiglas cubicles on a glass surface and allowed to acclimate for 30 min prior to the Hargreaves test. The light beam was focused on the bottom of the glass with the aid of an angled mirror and created an intense spot under the foot pad. Paw withdrawal latency was defined as the time required for the paw to show an abrupt withdrawal. In the absence of a response at 20 s, the stimulus was terminated and that latency was assigned.

### Drug treatment

All drugs were dissolved in DMSO-cremophor-saline (1:1:18), which was used as a vehicle control. CCI mice were randomly assigned to receive the ABHD6 inhibitor WWL70 or the vehicle control. Drugs were given intraperitoneally (i.p.) 3 h after surgery and then once daily until day 7. Animals were sacrificed on day 7, and the sciatic nerve, spinal cord, and DRG were collected for further analysis. To determine the cannabinoid receptor dependency, WWL70 (10 mg/kg) was co-administered with the CB1R antagonist AM281 (3 mg/kg, i.p.) or the CB2R antagonist AM630 (3 mg/kg, i.p.).

### qRT-PCR

On day 7 after CCI surgery, mice were euthanized, and the sciatic nerves, DRG, and spinal cords were dissected out. Total RNA was extracted from tissues using Trizol reagent (Sigma, St Louis, MO). Gene expression was assessed by SYBR green-based qRT-PCR. RNA (1 μg) was reverse transcribed to cDNA. Fifty nanograms of cDNA was added to the qPCR reaction containing 1× fast universal PCR master mix (Applied Biosystems, Grand Island, NY), and 100 nM of each primer was analyzed in a CFX96™ Real-Time System (Bio-Rad, Hercules, CA). Amplification was performed at 95 °C for 15 s, 75 °C for 1 min, and 60 °C for 30 s for 40 cycles, followed by a melting point determination or dissociation curves. The expression level of each gene was indicated by the number of cycles needed for the cDNA amplification to reach a threshold. Relative levels of gene expression were determined by the 2^−ΔCt^ method normalized to the expression of GAPDH. All primers used in this study are listed in Table 1.

**Table 1:** Forward and reverse sequences of the primers

Genes	Forward	Reverse
CCL2 (GI:148877898)	5′- cagcaagatgtcccaatga-3′	5′- tctggacccattccttcttg-3′
CCR2 (GI:187953012)	5′- attctccacaccctgtttcg-3′	5′- gattcctggaaggtggtcaa-3′
IL-4 (GI:341416)	5′- cctcacagcaacgaagaaca-3′	5′- atcgaaaagcccgaaagagt-3′
IL-1β (GI:15030320)	5′- gcaactgttcctgaactcaact-3′	5′- atcttttggggtccgtcaact-3′
IL-6 (GI:124376265)	5′- ccggagaggagacttcacag-3′	5′- cagaattgccattgcacaac-3′
TNF-α (GI:518831586)	5′- ccctcacactagatcatcttct-3′	5′- gctacgacgtgggctacag-3′
NGF (GI:53364)	5′- ctccacccacctcttcagac-3′	5′- cactgagaactcccccatgt-3′
COX2 (GI:31127109)	5′- gtggaaaaacctcgtccaga-3′	5′- gctcggcttccagtattgag-3′
PGES2 (GI:260763899)	5′- acttccactccctgccctat-3′	5′- gttgcaagctgtctccttcc-3′
EP2 (GI:13529403)	5′- atgctcctgctgcttatcgt-3′	5′- agggcctcttaggctactgc-3′
EP4 (GI:14290509)	5′- ccatcgccacatacatgaag-3′	5′- ctcatggcacagatgatgct-3′

### Immunostaining

Animals were euthanized using a combination of ketamine and xylazine (90 mg/kg ketamine/10 mg/kg xylazine in a volume of 10 μl/g body weight, i.p.) and intracardially perfused with ice-cold 1× PBS followed by 4% paraformaldehyde in 1× PBS. The spinal cord, DRG, and sciatic nerve were dissected out and post-fixed in 4% paraformaldehyde at 4 °C overnight. The tissues were cryoprotected in 30% sucrose/1× PBS at 4 °C until sinking, embedded in Tissue Tek OCT and stored at − 80 °C until use. Transverse sections of the lumbar spinal cord were cut at 14 μm, and transverse section of DRG and longitudinal section of sciatic nerve were cut at 10 μm by cryostat (Leica CM1900, Bannockburn, IL) and mounted onto Superfrost Plus slides for immunostaining. Primary antibodies included anti-goat Iba1 (1:300; Cat# ab48004, Abcam, Cambridge, MA), anti-rat F4/80 (1:400; Cat# 14–4801-82, eBioscience, San Diego, CA), anti-mouse GFAP (1:500; Cat# 3670, Cell Signaling, Danvers, MA), and anti-rabbit phosphorylated NF-kB (1:100; Cat# 65538, Full moon Biosystem, Sunnyvale, CA). Briefly, the slides were washed twice with 1× PBS and blocked in 1% donkey serum/1× PBS/0.3% Triton X-100 at room temperature for 30 min, followed by incubation with the respective primary antibody at 4 °C overnight. The slides were then washed with 1× PBS containing 0.2% Triton X-100 and incubated for 1 h at room temperature with Alexa fluor 488 or Alexa fluor 594 conjugated donkey anti-rabbit, -goat, -mouse, or -rat secondary antibodies (1:750; Thermo Fisher Scientific, Waltham, MA). The slides were again washed with 1× PBS twice and covered with Fluoroshield mounting medium with DAPI. Immunofluorescence images were obtained with a fluorescence microscope (Nikon Eclipse TE-2000U). The cells with both DAPI and expected fluorescence were defined as positively stained cells. All immunofluorescence data were obtained in a minimum of 5–7 serial sections from the lumbar spinal cord, DRG, and sciatic nerve of each animal. Immunoreactive positive cells for GFAP, Iba1, F4/80, and p-NF-κB were counted and expressed as mean cell numbers per square millimeter. Negative controls were routinely performed in which the primary antibodies were omitted.

### Enzyme-linked immunosorbent assay (ELISA)

Protein levels of IL-1β and CCL2, and the nuclei NF-κB binding activity were measured by ELISA. For the assay of IL-1β and CCL2, the fresh ipsilateral sciatic nerve tissues 7 days after surgery were solubilized with extraction buffer (100 mM Tris pH 7.4, 150 mM NaCl, 1mM EGTA, 1mM EDTA, 1% Triton X-100 0.5%, 0.5% sodium deoxycholate, and complete Mini protease inhibitor cocktail). The ELISA kits were precoated with anti-mouse IL-1β or CCL2 antibodies. Each sample (1 μg) was run in duplicate to quantify the protein levels of IL-1β and CCL2 based on the manufacturer’s protocol (Thermo Fisher, Waltham, MA). For the measurement of NF-κB activity, the NF-κB p65 transcription factor assay kit from Abcam (Cat# ab133112, Cambridge, MA) was used. The nuclear extracts from the fresh spinal cords of CCI mice 7 days after surgery were obtained with NE-PER nuclear extraction kit (Thermo Fisher, Waltham). Briefly, about 10 mg of the spinal cord tissues were homogenized in the CER I buffer provided in the kit and incubated on ice for 10 min. CER II buffer was then added to the tubes containing homogenized tissue and centrifuged for 5 min at maximal speed (16,000 g). After removing the supernatant, the pellet was suspended in ice-cold nuclear extraction reagent (provided in the kit) and centrifuged at maximal speed for 10 min. The supernatant (nuclear extract) was collected and analyzed for NF-κB activity assay following the manufacturer’s protocol (Cat# ab133112, Abcam, Cambridge, MA).

### Western blot

The whole cell lysates from sciatic nerves of CCI mice 7 days after surgery were prepared with RIPA buffer (10 mM Tris-HCL pH 7.4, 30 mM NaCl, 1 mM EDTA, 1% Nonidet P-40, supplemented with 1 mM Na_3_VO_4_, 1 µg/ml leupeptin, 1 µg/ml pepstatin A, 1 µg/ml aprotinin and 1 mM PMSF). Protein concentration was determined for each sample, and equal amount of proteins were run on 4–15% SDS-PAGE. Thereafter, proteins were transferred onto nitrocellulose membranes, blocked for 1 h at RT with 5% non-fat milk, and incubated overnight at 4 °C with the primary rabbit antibodies against phosphorylated cPLA_2_ (1:300; Cat# 2831, Cell Signaling) and total cPLA_2_ (1:1000; Cat# 2832, Cell Signaling). After probing with goat anti-rabbit IgG (H + L)-HRP-conjugated secondary antibody (1:2500; Bio-Rad, Hercules, CA) for 1 h at 25 °C, protein bands were detected using the Supersignal West Pico Chemiluminescence (Thermo Fisher, Waltham, MA). Membranes were subsequently probed for β-actin expression to serve as a loading control. Images were acquired and analyzed with a Chemidoc Touch image system (BioRad).

### PGE_2_ assay in sciatic nerve

On day 7 post-CCI, mouse sciatic nerve was obtained and homogenized with 40 μl of 0.02% trifluoroacetic acid (TFA) and 100 μl of acetonitrile on ice. In order to extract maximal lipid, 140 μl of tissue homogenate was dispersed in 1 ml acetonitrile by vortex and left at 4 °C overnight. On the second day, the homogenate-acetonitrile mixture was centrifuged at 2000 g for 5 min to remove the debris and the supernatant was transferred to a silanized glass tube. The supernatant was evaporated under the nitrogen gas streaming in a water bath (approx. 35 °C) and then reconstituted with acetonitrile. The levels of PGE_2_ in the lipid extract were measured with a PGE_2_ enzyme immunoassay (EIA) kit following the manufacturer’s protocol (Cayman Chemical, Ann Arbor, MI).

### LC-MS/MS analysis for 2-AG and AA

The sciatic nerve tissue obtained from mice 7 days post-CCI was homogenized with 40 μl of 0.02% TFA, 250 μl of acetonitrile, and 250 picomoles of 2-AG-d5 (Cayman Chemical) using a Potter homogenizer under 4 °C environment. The homogenate was dissolved completely in 2.5 ml acetonitrile by vortex and kept at 4 °C overnight. The homogenate was subjected to centrifugation at 2000 g × 5 min to remove the debris, and then, the supernatant was evaporated under the nitrogen gas streaming in a water bath (approx. 35 °C). The lipid was re-suspended with 100 μl of acetonitrile and stored at − 80 °C until use.

An HPLC system (1200 Series, Agilent Technologies, Santa Clara, CA) was used with a reverse phase guard column (Wide Pore C18 (ODS), 4 × 2 mm ID; Phenomenex, Torrance, CA), and the column (Sephasil Peptide C18, 5 μ, ST, 100 × 4.6 mm ID; Pharmacia Biotech, Piscataway, NJ) was maintained at 40 °C. The mobile phase was composed of solvent A (0.2% formic acid in water) and solvent B (0.2% formic acid in methanol), and the following gradient was used: 62% A/38% B isocratic for 30 s., ramp to 90% B in 60 s., isocratic at 90% B for 18.5 min., ramp back to 62% A/38% B in 60 s., and re-equilibrate at 62% A/38% B for 8 min. The flow rate was 0.4 ml/min. The HPLC output was directed into the TurboV electrospray ionization (ESI) source of a Q-Trap 4000 mass spectrometer (AB Sciex, Framingham, MA). The injection volume was 20 μl. LC-MS/MS analysis was performed in a positive mode with the ion source temperature of 600 °C, a spray voltage of 5.5 kV, and a declustering potential of 45 V. Multiple reactions monitoring (MRM) was performed on the transitions *m*/*z* 379→287 for 2-AG, 384→292 for 2-AG-d5, and 305→93 for AA. The concentrations of 2-AG and AA were determined by calculating the corresponding peak area ratio to the internal standard (IS) using a linear fit weighting to the calibration curve.

### Statistical analysis

Data were analyzed for statistical significance by using an unpaired two-tailed *t* test (comparison of two data sets) or with ordinary one-way or a two-way analysis of variance (ANOVA, comparison of multiple data sets). All experiments were repeated at least three times. The data are expressed as mean ± SEM. A significant difference was determined as *p* < 0.05.

## Results

### CCI-induced mechanical allodynia and thermal hyperalgesia were attenuated by administration of WWL70

**Fig. 1:**
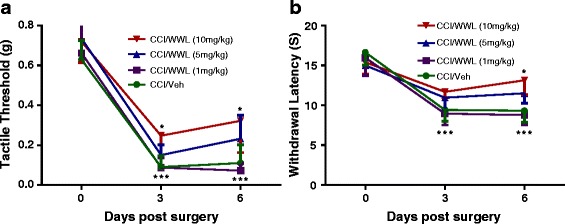
WWL70 dose dependently alleviated neuropathic pain in CCI mice. At days 0, 3, and 6 after surgery, mechanical thresholds were evaluated by the “Up-Down” method using von Frey filaments (**a**) and thermal withdrawal latency was determined by the Hargreaves test (**b**). Treatment with WWL70 at 10 mg/kg significantly increased thermal withdrawal latency and mechanical thresholds compared to the vehicle group. However, at lower doses, WWL70 had no effect, although there was a trend towards the increased tactile threshold and withdrawal latency when treated with 5 mg/kg. *p < 0.05 was obtained when the WWL70 treated group was compared to the CCI vehicle group; ***p < 0.001 compared to baseline (mean ± S.E.M., *n* = 8/group)

Eight to 10-week-old male C57BL/6 mice were subjected to CCI surgery and treated with various doses of WWL70 (1, 5, and 10 mg/kg). Nociceptive behavior was assessed before surgery (day 0) and at days 3 and 6 post-surgery. Compared to baseline, the CCI group displayed significant mechanical allodynia and thermal hyperalgesia in their ipsilateral paws (*p* < 0.001) at 3 and 6 days post-CCI (Fig. 1a, b). Treatment with 10 mg/kg WWL70 after CCI resulted in a significant suppression of nociceptive behavior compared to the CCI vehicle group (Fig. 1a, b). The average tactile threshold (*g*) was increased from 0.09 to 0.24 (*p* < 0.05) on day 3 and from 0.11 to 0.32 on day 6 (*p* < 0.05) indicative of decreased mechanical allodynia. Similarly, the WWL70 (10 mg/kg) treated mice showed reduced thermal hyperalgesia with a longer withdrawal latency from an average of 9.42 to 11.69 s on day 3, and 9.31 to 13.14 s on day 6 (*p* < 0.05) after CCI. Mice treated with 5 mg/kg WWL70 also showed an increased tactile threshold (Fig. 1a) and longer withdrawal latency (Fig. 1b), but were not significantly different from mice in the CCI vehicle group. Mice treated with 1 mg/kg WWL70 had the same nociceptive symptoms as the CCI vehicle mice (Fig. 1a, b).

### Alleviated neuropathic pain by WWL70 was independent of the cannabinoid receptor activation

**Fig. 2:**
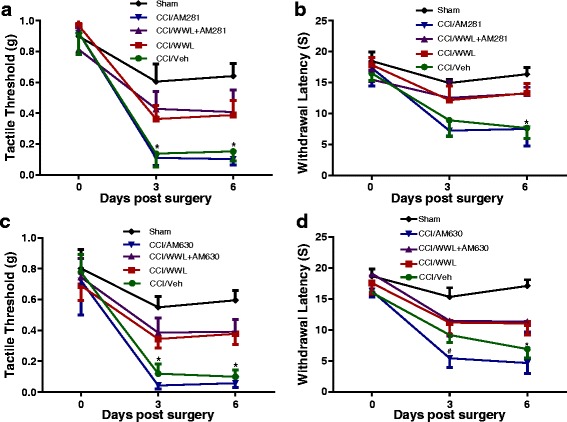
The anti-allodynic and anti-hyperalgesic effects of WWL70 in CCI mice were independent of cannabinoid receptor activation. Co-administration of WWL70 (10 mg/kg) with either CB1R antagonist AM281 (3 mg/kg) (**a**, **b**) or CB2R receptor antagonist AM630 (3 mg/kg) (**c**, **d**) in the CCI mice resulted in similar anti-allodynic and anti-hyperalgesia effects compared to the WWL70 alone treatment group. Treatment with AM630 itself exhibited an exaggerated thermal hyperalgesia compared to the CCI vehicle group on day 3. #*p* < 0.05 (**d**). No changes were seen by AM281 treatment alone (**b**). *p < 0.05 was obtained when the CCI vehicle group was compared to the WWL70, WWL70 plus AM281, and WWL70 plus AM630 treatment groups (**a**–**d**; mean ± S.E.M., *n* = 7/group)

We have recently reported that systematic administration of WWL70 at 10 mg/kg significantly inhibits the activity of ABHD6 and elevates 2-AG levels in the experimental autoimmune encephalomyelitis (EAE) mouse brain and spinal cord [21]. Blockage of endocannabinoid degradation has been shown to decrease mechanical and cold allodynia in CCI mice [15, 29]. To examine whether the inhibitory effect of WWL70 on neuropathic pain is mediated through the cannabinoid signaling pathway, the CB1R antagonist AM281 (3 mg/kg) or the CB2R antagonist AM630 (3 mg/kg) was administered to the CCI mice either alone or in combination with WWL70 (10 mg/kg). CCI mice treated with the CB1R antagonist AM281 alone exhibited a similar tactile threshold (Fig. 2a) and withdrawal latency (Fig. 2b) to those in the vehicle group. Addition of AM281 did not alter the inhibitory effect of WWL70 on mechanical allodynia and thermal hyperalgesia (Fig. 2a, b), suggesting that the therapeutic effect of WWL70 was not attributable to CB1R activation. Similarly, the inhibitory effect of WWL70 on CCI-induced mechanical allodynia and thermal hyperalgesia was also not affected by the CB2R antagonist AM630 (Fig. 2c, d). Notably, treatment with AM630 alone tended to further reduce the tactile threshold and resulted in a significant decrease in withdrawal latency on day 3 when compared to the CCI vehicle group. These data indicate that blocking the CB2R can exaggerate CCI-induced neuropathic pain (Fig. 2c, d).

### WWL70 reduced inflammatory response in the ipsilateral spinal cord, DRG, and sciatic nerve

**Fig. 3:**
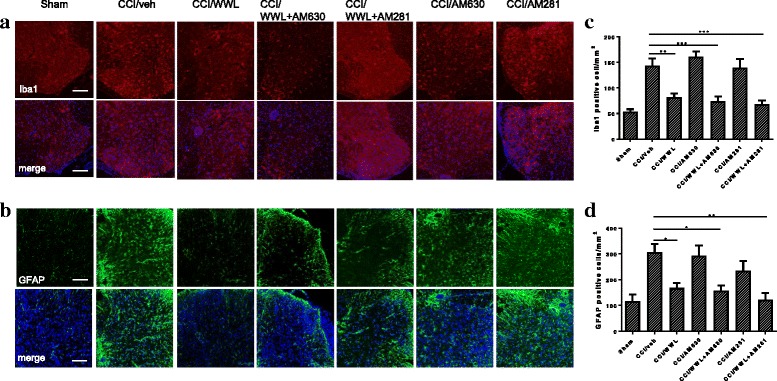
WWL70 treatment reduced microglia and astrocyte activation in the ipsilateral dorsal horn of mouse lumbar spinal cord. At 7 days post-injury, the Iba1-positive microglia/macrophages (**a**) and GFAP-positive astrocytes (**b**) were dramatically increased in CCI vehicle group and reduced in mice treated with WWL70 (10 mg/kg). Combined treatment with WWL70 (10 mg/kg) and AM630 (3 mg/kg) or AM281 (3 mg/kg) exhibited similarly reduced Iba1- and GFAP-positive cells in the spinal cord compared to the CCI vehicle group. Treatment with AM281 or AM630 alone did not alter the numbers of microglia and astrocytes. Quantification of the Iba1- and GFAP-positive stained cells (**c**, **d**). *p < 0.05, **p < 0.01, and ***p < 0.001 (mean ± S.E.M., *n* = 7/group). Scale bar = 250 μm. Merge represents co-localization of DAPI (blue) with Iba1 (red) or GFAP (green)

**Fig. 4:**
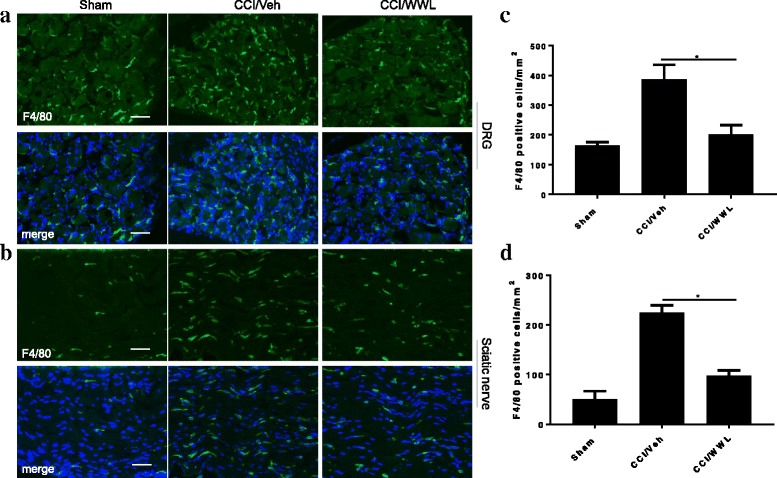
Reduced macrophage infiltration in CCI mouse ipsilateral DRG and sciatic nerve following WWL70 treatment. The numbers of F4/80 positive macrophages in the DRG (**a**) and sciatic nerve (**b**) in mice treated with WWL70 (10 mg/kg) after CCI were remarkably reduced compared to the CCI/vehicle group. (**c**, **d**) Quantification of the F4/80-positive cells in DRG and sciatic nerve (mean ± S.E.M., *n* = 8/group). Scale bar = 50 μm. Merge represents co-localization of DAPI (blue) with F4/80 (green)

Activation of microglia and astrocytes in the spinal cord dorsal horn and infiltration of macrophages to the peripheral nervous system play a pivotal role in the initiation and development of neuropathic pain [3]. At 7 days post-CCI, microglia and astrocytes showed dramatic activation in the ipsilateral spinal cord dorsal horn (Fig. 3a, b). The numbers of Iba1 and GFAP positive cells in the lumber spinal cord dorsal horn of mice after CCI that were administered vehicle only were 144 ± 39 cells/mm^2^ and 306 ± 44 cells/mm^2^, respectively (Fig. 3c, d). CCI mice treated with WWL70 (10 mg/kg) showed significantly fewer Iba1 (82 ± 18 cells/mm^2^) and GFAP (168 ± 40 cells/mm^2^) positive cells (Fig. 3c, d). To further determine whether the cannabinoid signaling pathway was involved in the action of WWL70, GFAP, and Iba1 positive cells were counted in the dorsal spinal cords of CCI mice treated with the combination of WWL70 with either the CB1R or CB2R antagonist (Fig. 3c, d). WWL70 co-treatment with AM630, the CB2R antagonist, did not alter the effect of WWL70 treatment alone, with a similar number of reactive microglia and astrocytes (Iba1^+^ 74 ± 24 cells/mm^2^ and GFAP^+^ 157 ± 43 cells/mm^2^) found in mice in this treatment group. Treatment with WWL70 and AM281, the CB1R antagonist, further reduced the number of microglia (68 ± 18 cells/mm^2^) and astrocytes (123 ± 50 cells/mm^2^) compared to the WWL70 alone treatment group. We then examined whether WWL70 administration affected macrophage infiltration in the ipsilateral sciatic nerve and DRG assessed by immunofluorescence labeling with F4/80, a marker of microglia/macrophages. F4/80 positive cells were significantly increased in the DRG and sciatic nerve after CCI (388 ± 49 and 225 ± 15 cells/mm^2^) compared to those in the sham group (164 ± 12 cells/mm^2^ in DRG and 51 ± 16 cells/mm^2^ in sciatic nerve) (Fig. 4a–d). WWL70 treatment dramatically reduced F4/80 positive cells in the DRG (201 ± 31 cells/mm^2^) (Fig. 4a, c) and the sciatic nerve (98 ± 10 cells/mm^2^) (Fig. 4b, d).

### WWL70 treatment suppressed the production of pro-inflammatory cytokines in the central and peripheral nervous system of CCI mice

**Fig. 5:**
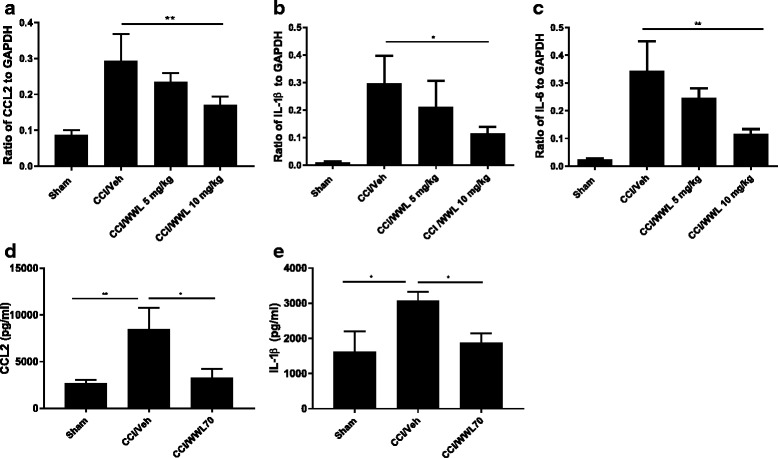
WWL70 treatment attenuated both mRNA and protein level of pro-inflammatory cytokines and chemokines in the ipsilateral sciatic nerve of mice after CCI. RNA isolated from sciatic nerves removed from mice 7 days post-CCI surgery were subjected to qRT-PCR analysis. CCL2 (**a**), IL-1β (**b**), and IL-6 (**c**) expressions were significantly elevated after CCI and reduced by WWL70 at 10 mg/kg, but not at 5 mg/kg treatment. *p < 0.05 and **p < 0.01 CCI/WWL70 (10 mg/kg) compared to CCI/vehicle group (mean ± S.E.M., *n* = 10/group). The production of CCL2 (**d**) and IL-1β (**e**) in sciatic nerve was measured by ELISA and shown to be significantly increased in the vehicle group compared to the control group (*p < 0.05 and **p < 0.01) and dramatically reduced by WWL70 (10 mg/kg). (*p < 0.05, mean ± S.E.M., *n* = 5/group)

**Fig. 6:**
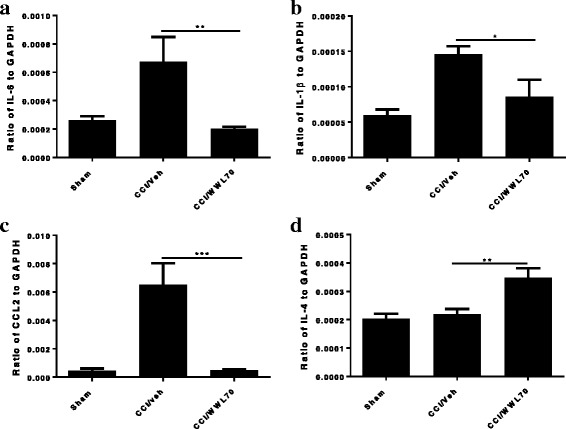
WWL70 treatment alleviated inflammatory response in CCI mouse spinal cord. The lumbar spinal cords 7 days post-CCI were dissected, RNA isolated, cDNA synthesized, and gene expression analyzed by qRT-PCR. Increased expression of IL-6 (**a**), IL-1β (**b**), and CCL2 (**c**) were observed in the CCI vehicle group and significantly decreased by WWL70 (10 mg/kg). (*p < 0.05, **p < 0.01, and ***p < 0.001 CCI/WWL70 compared to CCI/vehicle) (mean ± S.E.M., *n* = 10/group). WWL70 treatment after CCI also significantly increased IL-4 mRNA expression (**d**), **p < 0.01 (mean ± S.E.M., *n* = 10/group)

**Fig. 7:**
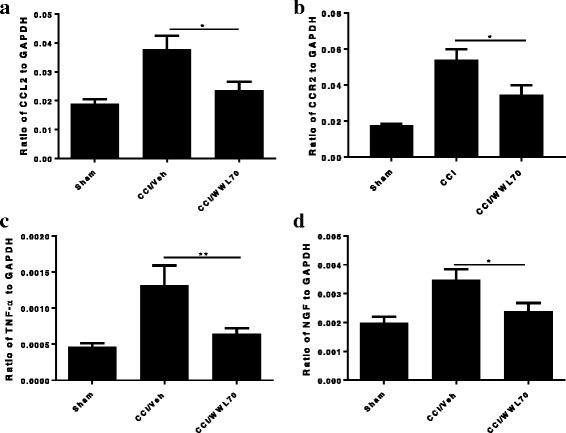
Elevated chemokines and cytokines in the ipsilateral DRG of CCI mice were downregulated by WWL70 treatment. RNA isolated from DRG taken from mice 7 days post-CCI was subjected to qRT-PCR analysis. The increased expression of CCL2 (**a**), CCR2 (**b**), and TNF-α (**c**) was detected in the CCI vehicle group and significantly suppressed by WWL70 (10 mg/kg). (*p < 0.05 and **p < 0.01 CCI/WWL70 compared to CCI/vehicle) (mean ± S.E.M., *n* = 10/group). NGF expression level was significantly increased in CCI vehicle group and attenuated by WWL70 administration (**d**). *p < 0.05 (mean ± S.E.M., *n* = 10/group)

The inflammatory response plays a critical role in neuropathic pain initiation and maintenance [2, 3]. The enhanced microglia proliferation in the spinal cord and the infiltration of macrophages into the DRG of the CCI mice contribute to the development of neuropathic pain by secreting pro-inflammatory cytokines such as TNF-α, IL-1β, and IL-6 [30]. We have previously found that the therapeutic effect of WWL70 in the mouse model of EAE correlates with the reduction of inflammatory cells and the pro-inflammatory cytokines [21]. To investigate the effect of WWL70 on the inflammatory response, we first measured the mRNA levels of several cytokines in the CCI mouse sciatic nerve. Treatment with WWL70 at 10 mg/kg significantly reduced the mRNA expression of CCL2, IL-1β, and IL-6 compared to the CCI vehicle groups (Fig. 5a–c). Although the expression of these cytokines was also attenuated by WWL70 treatment at 5 mg/kg, no statistical difference was reached (Fig. 5a–c). This result is in harmony with the dose-dependent effect of WWL70 on pain behavior (Fig. 1); therefore, 10 mg/kg of WWL70 was used in the rest of the experimental studies. To further verify the anti-inflammatory effect of WWL70, the protein levels of CCL2 and IL-1β in CCI mouse sciatic nerve were measured by ELISA. The levels of CCL2 in the CCI vehicle group were 8507 ± 2175 pg/ml, significantly greater than the sham group (2677 ± 388 pg/ml) and WWL70 treatment group (3266 ± 938 pg/ml) (Fig. 5d). Similarly, the protein levels of IL-1β were increased from 1630 ± 575 pg/ml pg/ml in sham group to 3072 ± 259 pg/ml in the vehicle group and reduced to 1800 ± 202 pg/ml in the WWL70 treatment group (Fig. 5e). Analysis of the lumbar spinal cord dorsal horn removed from mice 7 days after CCI also showed a significant increase of the mRNA expression of IL-6, IL-1β, and CCL2 (Fig. 6a–c), and this increase was greatly reduced by WWL70. Conversely, the expression of the anti-inflammatory cytokine IL-4 was elevated by WWL70 treatment (Fig. 6d). In the DRG of mice 7 days after CCI, we found a more than twofold increase in the expression of CCL2, CCR2, and TNF-α (Fig. 7a–c) compared with the sham mice. WWL70 treatment reduced this expression close to control levels. Nerve growth factor (NGF) is mainly synthesized in the DRG and transported through anterograde transport into the dorsal horn of the spinal cord where it is thought to participate in neuropathic pain development and transmission [31, 32]. CCI led to an elevation in NGF mRNA in the DRG at 7 days after surgery which was dramatically reduced by WWL70 (Fig. 7d).

### WWL70 attenuated phosphorylation of NF-κB in sciatic nerve and DRG and NF-κB activity in the spinal cord dorsal horn of CCI mice

**Fig. 8:**
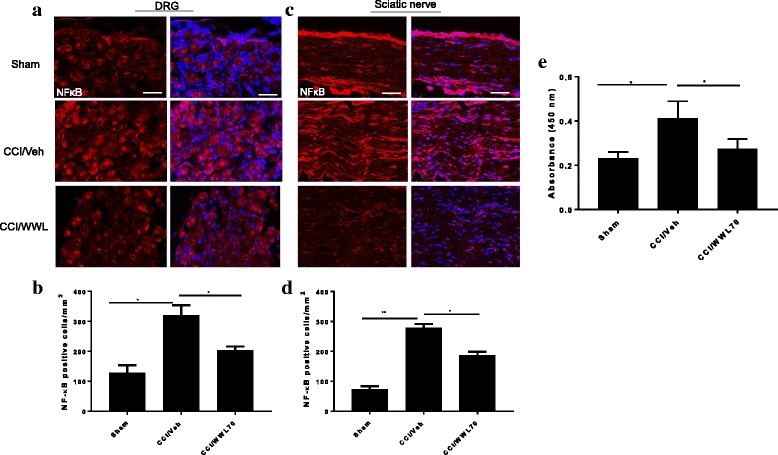
Treatment with WWL70 attenuated the phosphorylation of NF-κB in DRG and sciatic nerve of CCI mice and reduced NF-κB DNA binding activity in the lumbar spinal cord dorsal horn of CCI mice. The activation of NF-κB was detected by immunofluorescence staining with an antibody against phosphorylated NF-κB in ipsilateral DRG and sciatic nerve from CCI mice 7 days after surgery. Positive phosphorylated NF-kB staining in DRG (**a**) and sciatic nerve (**c**) was significantly greater than that in the sham group and dramatically reduced by WWL70 (10 mg/kg) treatment. The quantitation of NF-kB-positive stained cells was shown (**b**, **d**). *p < 0.05 and **p < 0.01 CCI/vehicle compared to the sham or CCI/WWL70-treated group (mean ± S.E.M., *n* = 8). Scale bar = 50 μm. Nuclear extract from the lumbar spinal cord dorsal horn of CCI mice was assessed for NF-κB DNA binding with a non-radioactive ELISA assay (**e**). Absorbance at 450 nm indicated that the activity of NF-κB in the sham group was smaller than that in the CCI/vehicle group. (*p < 0.05; mean ± S.E.M., *n* = 5/group). The O.D. value in the WWL70 treatment group was also significantly reduced when compared to the CCI/vehicle group. (*p < 0.05; mean ± S.E.M., *n* = 5/group)

It has been reported that activation of NF-κB is involved in the induction and maintenance of inflammatory pain, which can be weakened by the use of NF-κB inhibitors [33, 34]. Our previous study also demonstrated that WWL70 inhibited NF-κB transcriptional activity in BV2 cells and attenuated NF-κB phosphorylation in the EAE mouse spinal cord [21, 24]. To elucidate the potential mechanisms underlying the reduced pro-inflammatory cytokines in WWL70 treated CCI mice, we first examined NF-κB phosphorylation in the DRG and sciatic nerve. Compared to the sham control, tissues from sciatic nerve and DRG of the CCI vehicle group exhibited more phosphorylated NF-κB positive cells (Fig. 8). The average number of cells in the DRG and sciatic nerve that had positive phosphorylated NF-κB staining after CCI was 320 ± 59 cells/mm^2^ and 279 ± 17 cells/mm^2^, respectively, compared with 128 ± 44 cells/mm^2^ and 73 ± 14 cells/mm^2^, respectively, in sham mice (Fig. 8b, d). Treatment with WWL70 greatly reduced phosphorylated NF-κB positive cells in both DRG (204 ± 22 cells/mm^2^, Fig. 8b), and sciatic nerve (186 ± 18 cells/mm^2^, Fig. 8d). Next, we examined the NF-κB DNA binding activity using the nuclear extracts from the lumbar spinal cord dorsal horn of CCI mouse. NF-κB contained in the nuclear extract binds specifically to the NF-κB response element, which can be detected by ELISA. The amount of NF-κB binding was indicated by absorbance at 450 nm (OD_450_). Consistent with the increased phosphorylation of NF-κB in DRG and sciatic nerve, the NF-κB binding activity was increased in the CCI vehicle group compared to the sham group, but reversed significantly in WWL70-treated groups. The average OD_450_ values in the sham, CCI/vehicle, and CCI/WWL70-treated groups were 0.23, 0.41, and 0.27, respectively (Fig. 8e). These results are in agreement with our previous study [21, 24], suggesting that WWL70 can exert an inhibitory effect on NF-κB activation to downregulate the production of inflammatory cytokines following CCI.

### WWL70 suppressed eicosanoid metabolic pathway in injured sciatic nerve of CCI mice

**Fig. 9:**
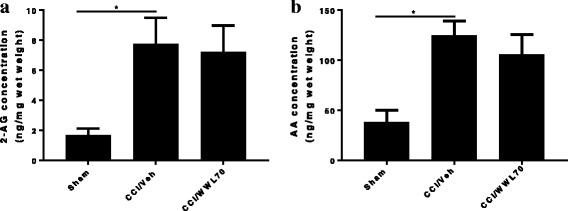
Treatment with WWL70 did not reduce the elevated levels of 2-AG and AA in CCI mouse sciatic nerve. Fresh sciatic nerve tissues from CCI mice 7 days after surgery were subjected to LS-MS/MS. The amount of 2-AG (**a**) and AA (**b**) was significantly increased in the vehicle group compared to the sham. (*p < 0.05; mean ± S.E.M., *n* = 7). WWL70 (10 mg/kg) treatment did not significantly reduce the increased production of 2-AG and AA elicited by CCI, although a slight reduction was observed for the latter

Recent studies have found that MAGL is a rate-limiting enzyme for the production of arachidonic acid (AA) and the subsequent prostaglandins in the mouse brain [35]. Consequently, inhibition of MAGL has anti-inflammatory and neuroprotective effects in animal models of Parkinson’s and Alzheimer’s disease by interfering with eicosanoid rather than cannabinoid signaling [36, 37]. To determine whether inhibition of ABHD6 can also affect the eicosanoid metabolism as we found in the EAE mouse model [21], the levels of 2-AG and AA in the sciatic nerve after CCI were measured by LS-MS/MS. At 7 days post-injury, there was a significant increase in the levels of 2-AG (8.85 ± 1.54 ng/kg) and AA (124.57 ± 14.40 ng/kg) in the injured sciatic nerve compared to the levels of 2-AG (1.68 ± 0.44 ng/kg) and AA (37.95 ± 11.83 ng/kg) in the sham animals. WWL70 treatment did not reduce the levels of 2-AG at this time point (8.23 ± 1.66 ng/kg), although there was a non-significant reduction in AA levels (105.76 ± 19.74 ng/kg) in sciatic nerve (Fig. 9). These results suggest that inhibition of ABHD6 may not directly control the levels of 2-AG and AA in the sciatic nerve of sham and CCI animals.

**Fig. 10:**
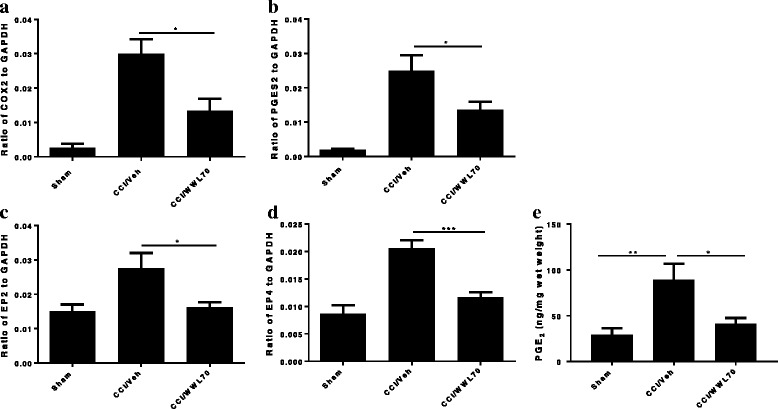
WWL70 treatment reduced PGE_2_ synthesis in CCI mouse sciatic nerve. The fresh sciatic nerves from mice 7 days post-CCI surgery were subjected to qRT-PCR analysis. The enzymes that contribute to the PGE_2_ synthesis COX-2 (**a**) and PGES2 (**b**) were greatly increased in CCI vehicle group and reduced by WWL70 (10 mg/kg). *p < 0.05. The increased expression of EP2 and EP4 receptor in the CCI vehicle mice was also attenuated by WWL70 treatment (**c**, **d**; *p < 0.05 and ***p < 0.001; mean ± S.E.M., *n* = 10). PGE_2_ production was measured by EIA assay. Significantly increased PGE_2_ in the vehicle group was remarkably blocked by WWL70 administration ((**e**), *p < 0.05 and **p < 0.01; mean ± S.E.M., *n* = 7)

Several studies have shown that CCI results in high levels of COX-2 and PGE_2_ in the injured peripheral nerve, suggesting a critical role for enhanced eicosanoid signaling in the initiation and maintenance of neuropathic pain [26, 38, 39]. To determine whether the reduction of neuropathic pain by WWL70 involved modulation of PGE_2_ and its synthetic enzymes, we examined mRNA expression of COX-1, COX-2, the PGE_2_ synthetic enzymes PGES1 and PGES2, and the PGE_2_ receptors EP1, EP2, EP3, and EP4 in sciatic nerve. In the injured sciatic nerve after CCI, there was a significant increase in the expression of COX2, PGES2, EP2, and EP4 genes. WWL70 treatment significantly reduced the expression of all these genes (Fig. 10a–d). There was no difference in the expression of COX-1, PGES1, EP1, and EP3 receptors in the sciatic nerve of injured compared to sham mice (data not shown). We then measured the levels of PGE_2_ in sciatic nerve by enzyme-linked immunoassay. Seven days post-CCI, we found a significant increase of PGE_2_ in the sciatic nerve (89.6 ± 17.2 ng/mg wet weight) compared to the sham control (29.1 ± 7.5 ng/mg). Treatment with WWL70 after CCI significantly reduced PGE_2_ levels (41.3 ± 6.2 ng/mg) (Fig. 10e).

**Fig. 11:**
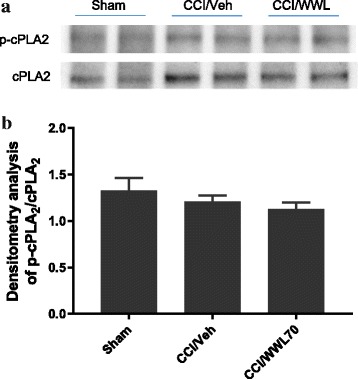
WWL70 treatment had no effect on cPLA_2_ phosphorylation in CCI mouse sciatic nerve. **a** Representative blot of two tissue lysates made from sciatic nerve removed from sham and CCI injured mice at 7 days after injury probed with antisera specifically recognizing p-cPLA_2_ or total cPLA_2_. **b** Quantification of western blot data showing the relative ratio of p-cPLA_2_/cPLA_2_ each normalized to β-actin (mean ± S.E.M., *n* = 4)

To further determine whether WWL70 treatment can also affect the activity of cPLA_2_, a critical enzyme for AA liberation and the subsequent production of prostaglandins [40], the expression of phosphorylated cPLA_2_ (p-cPLA_2_) and total cPLA_2_ in sciatic nerve 7 days after CCI was assessed by western blot (Fig. 11). The levels of p-cPLA_2_ were similar in control, vehicle, and WWL70 treated groups. Densitometry analysis of p-cPLA_2_ to cPLA_2_ in the western blot also indicated that the expression of p-cPLA_2_ was not altered, suggesting that cPLA_2_ does not participate in either CCI-induced neuropathic pain pathogenesis or the anti-inflammatory effect of WWL70.

## Discussion

Inhibition of the major 2-AG hydrolytic enzyme MAGL has been shown to alleviate inflammatory and neuropathic pain [11–13, 15], but it is unclear whether blockage of the activity of ABHD6, a minor 2-AG hydrolytic enzyme, is also effective for pain management. We have recently found that the ABHD6 inhibitor WWL70 has a neuroprotective effect in the EAE mouse model by modulating the endocannabinoid signaling and reducing the exaggerated inflammatory response. In this study, we revealed that WWL70 possessed a potent anti-nociceptive effect after CCI. Surprisingly, our data suggest that the therapeutic mechanism of WWL70 in neuropathic pain is due to its suppression of COX-2 and PGES2 expression leading to a reduction in PGE_2_ rather than through the activation of cannabinoid receptors.

In response to nerve injury, the resident immune cells, including mast cells, macrophages, and glial cells, are activated and contribute to the pain initiation by releasing nociceptive mediators and recruiting immune cells to the damaged nerve [30, 41–43]. It has been reported that fluorocitrate, which inhibits glia activation [44, 45], and minocycline, which selectively disrupts the activity of microglia, are able to alleviate allodynia/hyperalgesia [46–49]. These studies support the notion that glia activation is necessary for the initiation of the exaggerated response to noxious stimuli. Our previous study in the mouse model of EAE [21] has clearly demonstrated the inhibitory effect of WWL70 in microglia/macrophage infiltration. The increased activation of microglia and astrocytes in the DRG, sciatic nerve, and dorsal horn of the lumbar spinal cord of the CCI vehicle group indicates the importance of glial activation in CCI-induced neuropathic pain. Considering the inhibitory effect of WWL70 on microglial activation in our previous studies, we explored the possibility of WWL70 in the treatment of neuropathic pain. As anticipated, systemic administration of WWL70 to mice with CCI dramatically reduced microglia and astrocyte activation in the spinal cord dorsal horn and macrophage infiltration in the sciatic nerve and DRG. Moreover, the reduced activation of glial cells by WWL70 treatment after CCI further alleviated the release of pro-nociceptive mediators to facilitate pain relief. These results are consistent with several recent studies indicating spinal microgliosis and macrophage accumulation in DRG and sciatic nerve contribute to pain hypersensitivity after peripheral nerve injury [50, 51].

In addition to increased microglia/macrophage activation in CCI mice, we found a remarkable upregulation of chemokine C-C motif ligand 2 (CCL2, also known as MCP-1) mRNA expression in the DRG, sciatic nerve, and spinal cord, in agreement with several previous studies [52–54]. CCL2 secreted from DRG neurons can act on CCR2 expressed in microglia to initiate neuron-glia communication following peripheral nerve injury. Besides being expressed at the site of the injury, CCR2 is also present in the DRG and spinal cord, where macrophages and microglia release pro-inflammatory mediators that excite nociceptive neurons to increase pain sensitivity [53, 55, 56]. Mice with gene deletion of CCR2 exhibit reduced mechanical allodynia after partial ligation of the sciatic nerve [57]. Conversely, enhanced pain sensitivity was observed in mice with overexpression of CCL2 in astrocytes [58]. Therefore, control of CCL2/CCR2 signaling is able to benefit pain management. We found that CCL2 was significantly upregulated after CCI in the DRG, spinal cord dorsal horn, and sciatic nerve tissues. Moreover, we demonstrated that CCR2 was strongly induced in the DRG and moderately increased in the spinal cord dorsal horn in CCI mice. Together, these observations further verified the involvement of CCL2/CCR2 signaling in CCI-induced neuropathic pain. Our observation that the increased production of CCL2 and CCR2 was significantly reduced by WWL70 treatment suggests that the interference in CCL2/CCR2 signaling may account for the anti-nociceptive effect of WWL70.

In addition to the direct impact on pain initiation and propagation, CCL2 and its receptor CCR2 are important intracellular signaling molecules that regulate neural plasticity and inflammatory response [2]. After CCI, we detected a significant increase of IL-1β, TNF-α, and other pro-inflammatory cytokines or chemokines in the sciatic nerve, DRG, and lumbar spinal cord. The role of IL-1β in neuropathic pain development is clearly demonstrated by decreased hyperalgesia in IL-1β receptor knockout mice [59]. Endoneurial administration and topical application of TNF-α along with a restricted portion of the sciatic nerve can either augment the excitability of axons or sensitize the axon terminals to elicit hyperalgesia [60, 61]. In this study, we found the increased expression of IL-1β, TNF-α, and IL-6 in the injured sciatic nerve was significantly reduced by WWL70 treatment. The phosphorylation of NF-κB which can drive the upregulation of pro-inflammatory cytokines and chemokines in sciatic nerve, DRG, and dorsal horn of the spinal cord was also dramatically reduced in the WWL70-treated animals. These results are consistent with reports from several previous studies [52, 62–64] suggesting that the anti-nociceptive effect of WWL70 is attributable to its suppression of neuroinflammatory mediators.

Although inhibitors of MAGL have been shown to alleviate neuropathic pain via cannabinoid receptor-dependent mechanisms [15, 65], the degree to which cannabinoid signaling is involved in the anti-inflammatory and neuroprotective effects of ABHD6 inhibition is uncertain [19–21, 66]. We have previously demonstrated that the therapeutic effect of WWL70 is attributable to the activation of CB2R in the EAE mouse model [21]. However, here we find that the anti-nociceptive effect of WWL70 in the CCI mice is cannabinoid receptor independent. Consistent with the reported increase of 2-AG levels at the spinal and supraspinal levels in several models of neuropathic pain [67, 68], we found that at 7 days post-CCI, the levels of 2-AG in the injured sciatic nerve were elevated by fivefold, and this increase remained the same in the WWL70-treated animals. It is important to note that the level of AA in the injured sciatic nerve (124. 57 ± 14.40 ng/kg, Fig. 9b) is 14-fold higher than the level of 2-AG (8.85 ± 1.54 ng/kg, Fig. 9a), and this increase was also not affected by WWL70 treatment. This result suggests that the small pool of 2-AG derived from ABHD6 inhibition does not contribute to the bulk increase of 2-AG and AA in the sciatic nerve following injury. It also implies that the therapeutic effect of WWL70 is unlikely due to its modulation of cannabinoid signaling because of its limited inhibition of 2-AG hydrolysis. However, it is possible that the increased production of AA is derived from 2-AG hydrolysis by MAGL and ABHD12 [69] or membrane phospholipids by the action of secretory and calcium-independent PLA_2_ enzymes [70]. It has been recently reported that in the animal models of Parkinson and Alzheimer’s disease, the therapeutic effect of MAGL inhibitors is dependent on the reduced production of AA and prostaglandins rather than on the elevated cannabinoid signaling from high brain levels of 2-AG [35–37]. Similarly, we found that both 2-AG and AA can potentiate PGE_2_ production in lipopolysaccharide-activated microglia and the increased production of PGE_2_ can be blocked by WWL70 through cannabinoid receptor-independent mechanisms [24]. Several studies have revealed a persistent and chronic COX-2 and PGE_2_ upregulation in injured nerve tissues [71, 72], implying the importance of COX-2 and PGE_2_ in the development of neuropathic pain. Treatment with WWL70 significantly reduced the mRNA expression of COX-2, PGES2, EP2, and EP4 along with the production of PGE_2_ in the injured sciatic nerve. Our previous studies using BV2 microglia and EAE mouse spinal cord demonstrated that WWL70 may directly inhibit the activity of microsomal PGE synthase rather than the activity of COX-2 [21, 24]. Because AA production and the cPLA_2_ phosphorylation are not affected by WWL70, we speculate that WWL70 can interfere with the eicosanoid signaling cascade downstream of AA production, possibly through its inhibition of prostaglandin synthase and EP2 and EP4 receptor-mediated signaling. Therefore, the use of WWL70 may be advantageous for the treatment of neuropathic pain in comparison to COX inhibitors like nonsteroidal anti-inflammatory drugs (NSAIDs), which have significant gastrointestinal and cardiovascular side effects.

## Conclusions

This is the first study to examine the therapeutic role of ABHD6 inhibition in a rodent model of neuropathic pain. We have shown that the ABHD6 inhibitor WWL70 can significantly reduce thermal hyperalgesia and mechanical allodynia. The efficacy of WWL70 in this model is through reduction of PGE_2_ production in the injured sciatic nerve rather than inhibition of 2-AG hydrolysis. Our study therefore suggests that WWL70 may have therapeutic potential to treat a variety of inflammatory and neurological diseases in which activation of the COX-2-PGE_2_-EP axis contributes to the pathogenic mechanisms.

## Abbreviations

2-AG: 2-Arachidonoylglycerol
AA: Arachidonic acid
ABHD6: α/β-hydrolase domain 6
AEA: Anandamide
CB1R: Cannabinoid type 1 receptor
CB2R: Cannabinoid type 2 receptor
CCI: Chronic constriction injury
CCL2: Chemokine C-C motif ligand 2
COX-2: Cyclooxygenase-2
cPLA_2_: Cytosolic phospholipase A2
DRG: Dorsal root ganglion
EAE: Experimental autoimmune encephalomyelitis
EIA: Enzyme-linked immunoassay
ESI: Electrospray ionization
IL-1β: Interleukin-1β
IL-6: Interleukin-6
IS: Internal standard
LC-MS/MS: Liquid chromatography coupled with tandem mass spectrometry
MAGL: Monoacylglycerol lipase
MRM: Multiple reactions monitoring
NGF: Nerve growth factor
PGE_2_: Prostaglandin E_2_
PGES: Prostaglandin E_2_ synthase
TFA: Trifluoroacetic acid
